# Multiclass Classification of Cardiac Arrhythmia Using Improved Feature Selection and SVM Invariants

**DOI:** 10.1155/2018/7310496

**Published:** 2018-03-05

**Authors:** Anam Mustaqeem, Syed Muhammad Anwar, Muahammad Majid

**Affiliations:** ^1^Software Engineering Department, University of Engineering and Technology, Taxila, Pakistan; ^2^Computer Engineering Department, University of Engineering and Technology, Taxila, Pakistan

## Abstract

Arrhythmia is considered a life-threatening disease causing serious health issues in patients, when left untreated. An early diagnosis of arrhythmias would be helpful in saving lives. This study is conducted to classify patients into one of the sixteen subclasses, among which one class represents absence of disease and the other fifteen classes represent electrocardiogram records of various subtypes of arrhythmias. The research is carried out on the dataset taken from the University of California at Irvine Machine Learning Data Repository. The dataset contains a large volume of feature dimensions which are reduced using wrapper based feature selection technique. For multiclass classification, support vector machine (SVM) based approaches including one-against-one (OAO), one-against-all (OAA), and error-correction code (ECC) are employed to detect the presence and absence of arrhythmias. The SVM method results are compared with other standard machine learning classifiers using varying parameters and the performance of the classifiers is evaluated using accuracy, kappa statistics, and root mean square error. The results show that OAO method of SVM outperforms all other classifiers by achieving an accuracy rate of 81.11% when used with 80/20 data split and 92.07% using 90/10 data split option.

## 1. Introduction

In today's world, people are suffering from various chronic diseases. Among them heart diseases are found to affect a large size of population. An early detection and accurate medical assistance to heart disease patients can save human lives as heart diseases can be life-threatening causing sudden death. The most widely used tool for diagnosing the function of heart is the electrocardiogram (ECG) recorded using electrodes places on the body, which produces a graphical pattern of the electrical impulses of heart [1]. ECG signals are normally made of P waves, T waves, and QRS complex. The significant parameters required for the examination of heart-patients are time duration, shape, and the relationship between P wave, QRS complex, T wave, and R-R interval. Any abrupt change in these parameters indicates an ailment of the heart that may occur due to a wide range of reasons [2].

Arrhythmia is a form of irregularity in heart rhythms and, in some cases, results in heart disease, which poses serious threats to human lives. It should be diagnosed and treated as early as possible to reduce the risk of sudden death, since if left untreated it can also lead to a heart attack. Arrhythmia is a type of disease that disturbs the smooth rhythm of heart's electrical system and causes the heart to beat either too slow or too fast, to race, and to skip beats and causes nonsequential movement of heart signals. Generally, arrhythmia is identified and analyzed from an ECG recording along with the symptoms such as insufficient pumping of blood from heart, shortness of breath, fatigue, chest pain, and unconsciousness. Therefore, arrhythmia shows an abrupt and atypical ECG signal [3]. Arrhythmias are generally divided into two broad categories, that is, bradycardia and tachycardia. Bradycardia causes the heart to beat too slow, which is usually below the rate of 60 beats per minute (bpm), while tachycardia makes the heart beat faster which could go up to 100 bpm [4].

With the advent of numerous remote healthcare systems for cardiac patients, the importance of an efficient, intelligent, and robust arrhythmia classification system is being largely acknowledged. For developing an accurate diagnostic system, various machine learning techniques have been applied in the past to improve the accuracy of cardiac arrhythmia classification from ECG signals [5, 6]. The selection of an appropriate technique for arrhythmia classification is a complex task as it depends on the context of the application, data analysis, earlier experiences, and the requirements of the specified patients.

In this paper, we have proposed an efficient system, which can classify ECG records into normal and diseased classes, that is, differentiating between the existence and absence of arrhythmia. The dataset has been taken from University of California at Irvine (UCI) machine learning repository and multiclass classification is applied to classify the records into one of the 16 given classes. The dataset contains a large feature set which is reduced using an improved feature selection technique named as wrapper method. The proposed wrapper method is built on a random forest algorithm to select the most significant features from the given dataset [7]. The selected subset of features then undergoes a preprocessing step to introduce a uniformity in the distribution of data. Since support vector machine (SVM) is recognized to have the benefit of providing a notable performance in classification phase [8], three well-known and broadly used SVM methods are used in this research for classification of cardiac arrhythmia. A twin SVM (TSVM) is a multiclassification approach and is also used to test results on the arrhythmia dataset. TSVM is a new and unique machine learning approach, which aims at identifying two nonparallel planes for every class label. To accomplish the task, a pair of small sized quadratic problems is needed to be resolved rather than one larger problem [9].* K*-nearest neighbors (KNN), multilayer perceptron (MLP), the Naïve Bayes (NB), and random forest (RF) have also been trained and tested on the standard dataset to improve the accuracy and a comparison is done with the state-of-the-art systems showing an improvement in the classification accuracy. The main contributions of this paper are as follows:

1. An improved wrapper feature selection method built around random forest classifier for selecting the most relevant features is proposed.
2. SVM methods are implemented using the selected parameters to obtain a high multiclass classification accuracy on UCI-arrhythmia dataset.

This paper is organized into five sections. A discussion on previously designed and implemented arrhythmia classification models is given in Section 2. The detailed discussion of wrapper method for feature selection, data preprocessing, steps, and multiclass SVM based classification approaches is given in Section 3.  Section 4 presents the details of dataset, performance measures, and experimental evaluations using various parameters and, finally, the paper is concluded in Section 5.

## 2. Arrhythmia Classification Models

Many methods have been proposed to develop an intelligent classification model for arrhythmia detection. A learning vector quantization based neural network has been applied on the ECG dataset to classify cardiac arrhythmia patients. The features are reduced using principal component analysis followed by six neural networks to classify instants as normal or having arrhythmia [10]. Another research is carried out using automated artificial neural networks to classify arrhythmia patients using a standard 12-lead ECG data recording. The missing data is handled by replacing attribute values with the closest value from the concerned class. After replacing missing values, a multilayer perceptron with static backpropagation method is used for classification of arrhythmia [11]. Similar work is done using a generalized feed forward neural network [12], MLP with one-against-all method [13], Bayesian artificial neural networks [14], and modular neural networks [15] for classifying cardiac arrhythmia into 16 different classes. A new approach for cardiac arrhythmia classification is proposed in [16], which uses correlation based feature selection technique for selecting the most relevant features from the UCI ECG dataset, and incremental backpropagation neural network along with Levenberg–Marquardt is employed for an early and precise detection of arrhythmia.

Decision trees have also been used for successful classification of cardiac arrhythmia to design a computer assisted diagnosis system. These diagnosis and decision support systems can be helpful for physicians in diagnosing the disease efficiently and reducing the workload at the end of hospitals. A random forest ensemble method based on resampling strategy has been proposed to improve the classification systems designed for arrhythmia detection [17]. In [18], various machine learning algorithms including neural networks, decision trees, random forest, gradient boosting, and SVM are used for arrhythmia classification after applying rigorous preprocessing and feature selection techniques on ECG data. Similarly, OneR, J48, Naïve Bayes, SVM, logistic regression, KNN, random forest, and decision trees have been applied on ECG medical dataset to classify arrhythmia into 16 different classes [19–21]. Significant work on ECG dataset is conducted presenting SVM based methods for detecting arrhythmia with selection of significant features using principal component analysis [22, 23]. An efficient model for classification of arrhythmia patients is proposed, which uses SVM and KNN for training the model, and an improved accuracy measure is achieved using a combination of* F*-score and sequential forward search (SFS) for selection of features [24].

Feature selection techniques have their utmost significance in data mining, machine learning, and pattern recognition, especially for large datasets. A novel feature selection procedure based on dynamic mutual information is presented, which is only assessed on unsupervised data [25]. To validate the efficiency of the method, numerous experiments are conducted on UCI dataset using four typical classification algorithms. For selection of relevant features from ECG dataset, a novel ensemble based technique is proposed in [26] that selects feature subsets from the dataset and then classifiers are trained on each subset. The classifiers used are SVM, Naïve Bayes, and decision trees. Ensemble techniques are applied using a majority voting approach, which is applied on classification and feature selection error rates. The classification model of SVM can be enriched by allocating membership values for the given inputs. Therefore, a fuzzy SVM classifier combined with fuzzy theory is trained on UCI-arrhythmia dataset [27].

Traditional SVMs have higher computational complexity; a twin SVM method is proposed for binary classification problem to address this challenge [28]. Multiclassification is considered as a real world problem; therefore many variants of TSVM have been proposed [29]. A novel multiclass classification approach known as twin-KSVC (*K*-class Support Vector based Classification and Regression machine) has been proposed that takes advantage of the qualities of both* K*-SVCR and TSVM [30]. The purpose of this hybrid approach is to evaluate all training samples in a one-versus-one-versus-rest structure. Similarly, a* K*-nearest neighbor based weighted TSVM generalized as KWMTSVM has been proposed that employs the weight matrices in objective function to use the intraclass information [9].

In all these methods, the classification accuracy has still a room for improvement and the selection of the most significant features remains a challenging task. The critical nature of disease prognosis and diagnosis demands a highly accurate system to be used in clinical decision support systems. The proposed methodology addresses these concerns by selecting the most discriminating features using an improved feature selection method, which further helps in improving the classification performance.

## 3. Proposed Classification Model

**Figure 1:**
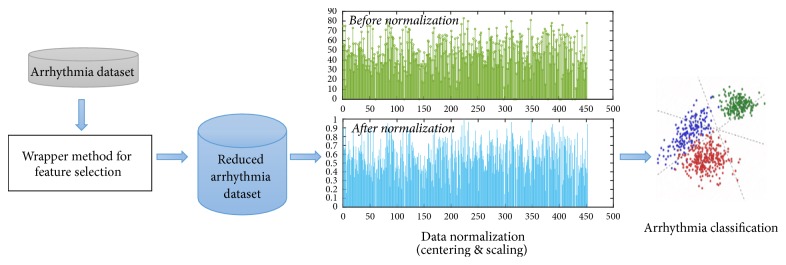
An overview of the steps involved in the proposed classification model.

In this paper, a new model is proposed for classifying arrhythmia patients using the ECG dataset taken from UCI machine learning repository. The proposed model first selects the most distinguishing features using an improved feature selection technique, a wrapper algorithm built around random forest classifier. After selecting the significant features, SVM based methods including one-against-one, one-against-all, and error-correction code are applied on the selected feature set to categorize the patients into sixteen subclasses of arrhythmia. The steps involved in the proposed model are shown in Figure 1.

### 3.1. Wrapper Method for Feature Selection

A large volume of dimensions in the dataset leads to poor classification accuracy especially in multiclass classification problem. Therefore, dimensionality reduction techniques are employed to reduce the dimensions and remove the redundant information from the dataset. Dimensionality reduction is a most commonly used preprocessing step in machine learning, data analysis, and data mining problems [31]. The dataset for arrhythmia patients from the UCI machine learning repository used in this research comprises a large feature set and instances. The task before classification is to reduce the data features, which play an important role in classifying the arrhythmia patients.

In this research, a wrapper method for feature selection (WFS) is used [32], which selects the most relevant features and filters out the other remaining attributes. The categorical features, which include binary values in the form of 0 and 1, are removed to reduce the original feature space as they have no contribution to the classification process. The feature selection algorithm used in this study is built around the RF classifier in the form of a wrapper, where the classifier is considered as a black box taking inputs and producing ranked output in terms of feature set. RF is a part of the knowledge based ensemble method in which classification process is carried out by a majority voting technique. RF is an ensemble method and therefore it is a combination of various weak classifiers, most of which form the decision trees. The voting of all weak classifiers in terms of the resultant output or decision is encountered to reach a final decision for output class [33]. These weak decision trees are executed independently on a set of bagging records of the dataset and the results are computed to reach a final decision. The measure for feature importance is obtained in terms of the average classification accuracy loss or error rate, which occurs during a random permutation of the attributes and is computed for all weak decision trees built in the random forest and, finally, an average error rate and the standard deviation are calculated.

**Figure 2:**
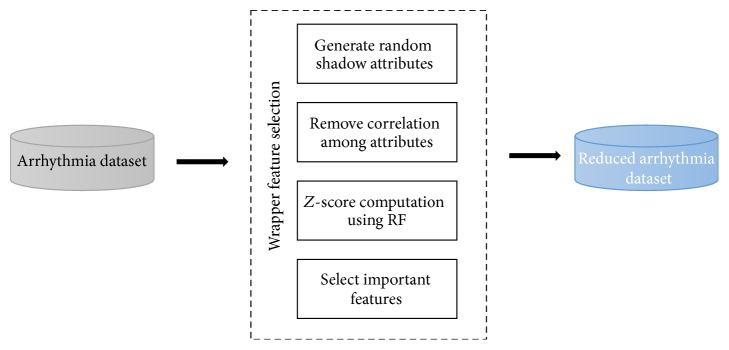
The steps involved in WFS method.

The steps involved in WFS are shown in Figure 2. The first step involves the generation of shadow attributes for all the features in the dataset to create shuffles and replicas. The purpose of adding replicas is to introduce randomness in the selected dataset. In this step, at least five copies of each data instance are created, which results in an increase in the number of records to a total of 2260. Once the data is extended, a knowledgeable ensemble based RF classifier is trained on the new data instances. After training, *z*-score is computed to measure the importance of features in terms of error rate [34]. The *z*-score represents the deviation of a data instance from the mean by computing the standard deviation of data instances that lies immediately above and below the mean. Hence, the *z*-score considers variation of the decreasing error rate among all the decision trees in the RF. The *z*-score is calculated by using

(1) $$\begin{array}{c} z_{i}=\frac{x_{i}-\overset{-}{x}}{s}, \end{array}$$

where $x_{i}-\overset{-}{x}$ shows the average error rate and *s* is the standard deviation of the data instances.

The WFS algorithm randomly generates shuffled shadow attributes. The computed *z*-score has no concern with the statistical significance of feature importance due to the normal distribution plot of *z*-score. RF is implemented in iterations to evaluate and compare the importance of feature set. The maximum value of *z*-score for the shadow attribute is picked and all other values are compared with this *z*-score to check the importance of features. Features which are less important based on the *z*-score are removed, while other significant features are used in the classification process. Once the importance of all the features is evaluated, the WFS algorithm terminates. The proposed WFS algorithm selected 94 important features from the feature space, which undergoes the data-preprocessing phase.

### 3.2. Data Preprocessing

Those features having large numeric values can have more impact on classification accuracy than the features having a smaller numeric range. The feature values in the dataset used in this study have a wide numeric range for different attributes. In such cases, the impact of features with a higher numeric range can be greater than other features. To overcome the impact of response variable for these features, data normalization [35] is performed. The main objective of data normalization is to improve the performance by limiting the impact of features with large data values on classification model. For data normalization, a centering and scaling technique is adopted for the original dataset values, which improves numeric stability of the proposed system. Centering transformation is applied to reduce the mean value of features, such that it becomes zero. For a set of observations *x*, such that *x* = {*x*_1_, *x*_2_, *x*_3_,…, *x*_*n*_}, after performing centering we obtain a new set of transformed observations given as

(2) $$\begin{array}{c} {cen}_{x_{i}}=\left\{\;x_{i}-\mu \left(\;x\;\right)\;\right\}, \end{array}$$

where cen_*x*_ = {cen_*x*_1_, cen_*x*_2_, cen_*x*_3_,…, cen_*x*_*n*_}, *n* is the total number of observations, and the resulting mean *μ*(cen_*x*_) equals 0.

After centering, a scaling transformation is applied such that cen_*x*_ has a standard deviation of one. The scaling transformation is applied by dividing each observation in cen_*x*_ by the standard deviation of all samples in set *x* and is defined as

(3) $$\begin{array}{c} sc\_x_{i}=\frac{{cen\_x}_{i}}{\sigma \left(x\right)}, \end{array}$$

such that *σ*(sc_*x*) equals 1.

### 3.3. Arrhythmia Classification

The preprocessed data is passed onto the next phase where classification is applied on the normalized data to detect the presence or absence of disease. Before classification, the normalized and filtered dataset is partitioned into training and testing sets. The model is trained by implementing three well-known methods of SVM including one-against-one (OAO), one-against-all (OAA), and error-correction code (ECC). The results are evaluated on the test data using accuracy, kappa statistics, and root mean square error as performance metrics.

The SVM classifier was initially designed to classify data instants into binary classes. The extension of SVM methods for multiclass classification problems has gained significant attention [36, 37]. There are two main methods for implementing a multiclass SVM classifier, including the pairwise coupling and one formulation for all. The formulation for solving multiclass problems in a single step needs variables that are proportional to the number of available classes. Therefore, for a multiclass classification problem using SVM, several numbers of binary classifiers are constructed to avoid constructing a bigger optimization problem. Computationally, multiclass problem is more expensive as it constructs several binary classifiers on the dataset as compared to a binary classifier that constructs a single classifier with the same amount of data [38]. A short description of the three SVM based classifiers including OAA, OAO, and ECC, which are used in this study, is given in the following subsections.

*One-against-All Method*. For a problem having *n* classes, the OAA approach goes for *n* number of SVM simulations [39]. The *i*th model of SVM is trained with rest of the correctly and incorrectly labelled training examples present in the *i*th class. The final output class of OAA approach is the one that has a high correspondence to the SVM model showing highest output performance. Therefore, for solving the SVM optimization problem using all training examples, the decision-making function for *n* models of SVM is given as

(4) $$\begin{array}{c} {\left(\;w^{1}\;\right)}^{T}\emptyset \left(\;x\;\right)+b^{1}\cdots {\left(\;w^{n}\;\right)}^{T}\emptyset \left(\;x\;\right)+b^{n}, \end{array}$$

where the training dataset *x* is mapped to a higher dimensional plane using the kernel function *∅*(*x*), (*w*^*n*^)^*T*^ represents a vector in the feature space of training set, and *b* is a scalar value. The input training set *x* is assigned to the class that gives higher decision function value. The correspondence function is given as

(5) $$\begin{array}{c} \text{class of }x=\arg\underset{i=1\cdots n}{\max}\left(\;{\left(\;w^{n}\;\right)}^{T}\emptyset \left(\;x\;\right)+b^{n}\;\right). \end{array}$$

*One-against-One Method*. OAO is another approach typically used for multiclass classification using SVM [39]. This approach constructs *n*(*n* − 1)/2 classifiers in such a way that each classifier is trained on the dataset taken from a pair of classes. Therefore, for training data taken from *i*th and *j*th classes, the binary classification problem is solved. After construction of classifiers, different methods can be employed for future testing such as the max-win strategy, which uses majority voting for selecting the output class of *x* and is computed as

(6) $$\begin{array}{c} f_{i,j}\left(\;x\;\right)=\left(\;{\left(\;{w_{i,j}}^{n}\;\right)}^{T}\emptyset \left(\;x\;\right)+{b_{i,j}}^{n}\;\right), \end{array}$$

where *f*_*i*,*j*_(*x*) = −*f*_*j*,*i*_(*x*)

(7) $$\begin{array}{c} \text{class of }x=\arg\underset{i=1\cdots n}{\max}\left(\;\overset{n}{\underset{j\neq i,j=1}{\sum }}sign\left(\;f_{i,j}\left(\;x\;\right)\;\right)\;\right). \end{array}$$

The output class of *x* is selected in such a way that the vote for *i*th class is incremented by 1 if *x* belongs to the *i*th class; otherwise the vote for *j*th class is incremented by 1. Finally, the class with the highest votes is predicted as the output class.

*Error-Correction Code*. This method combines several binary classifiers in such a way that error correction and decorrelations can be easily exploited [40]. This method constructs the decision function of *n* classifiers and takes the signs of these values. After computing decision function, it compares the Hamming distance between the output values of decision function and the instances of training dataset choosing the minimizer function as given below:

(8) $$\begin{array}{c} f\left(\;x\;\right)=\arg\underset{z=1\cdots r}{\min}\overset{n}{\underset{i=1}{\sum }}\left(\;\frac{1-sign\left(M_{zi}f_{i}\left(x\right)\right)}{2}\;\right), \end{array}$$

where *M* shows the training dataset and *r* is the size of dataset and *n* shows the number of classifiers. This method ensures that the resultant trained model yields good error-correction properties and for a minimum Hamming distance of *d*, the output model can correct (*d* − 1)/2 errors. The experimental details and results achieved are presented in the following subsections.

## 4. Experimental Details and Results

### 4.1. Dataset

This research is conducted on the arrhythmia dataset taken from the UCI machine learning repository. The dataset is composed of 452 samples classified into 16 different classes. The first class represents the normal cases, while the other 15 classes represent different types of arrhythmias including ischemic changes (coronary artery disease), old anterior myocardial infarction, old inferior myocardial infarction, sinus tachycardia, sinus bradycardia, ventricular premature contraction, supraventricular premature contraction, left bundle branch block, right bundle branch block, first-degree atrioventricular (AV) block, second-degree AV block, third-degree AV block, left ventricular hypertrophy, and atrial fibrillation. The dataset has a total of 279 attributes for each given sample where the first four attributes contain general information such as age, height, gender, and weight, while the rest of the attributes are extracted from the ECG signals recorded by a standard 12-lead recorder including the P, Q, R, S, and T waves information. As the dataset has a large set of features, feature selection is applied to select the most relevant and significant features containing useful information required for data classification. Similarly, in the preprocessing phase missing values for the selected attributes are handled by replacing them with the mean value of the column. After replacing missing values, the data is further normalized to bring uniformity in the dataset.

### 4.2. Performance Measures

The results of proposed arrhythmia classification system are evaluated using accuracy, kappa statistics, and root mean square error (RMSE). The classification accuracy is a widely used performance measure for comparing and analyzing the qualities of classification. However, if the distribution of classes among the data is skewed, then accuracy is not a good evaluation metric. Moreover, misclassification costs vary in severity especially in case of disease detection, whereas accuracy assumes equal costs for all errors including false positive and false negative errors. This can be problematic in real world scenarios; for example, misclassifying an arrhythmia patient to be healthy can cost the life of a patient. Still accuracy is the most commonly used evaluation measure along with other metrics including mean square error, kappa statistics, and receiver operating characteristics (ROC) curve [15]. The performance of the proposed classification model is accessed using these four measures. We have also evaluated the system using an overall accuracy of the system and an average per class. Accuracy can be calculated from the confusion matrix using

(9) $$\begin{array}{c} \text{Accuracy}=\frac{TP+TN}{TP+TN+FP+FN}, \end{array}$$

where TP (true positive) result shows a correct positive prediction of disease, TN (true negative) occurs when a correct decision is made regarding an absence of disease, FP (false positive) result shows the false prediction when a healthy patient is labelled as diseased, and FN (false negative) is the worst-case decision where a diseased patient is misclassified as a healthy patient. RMSE is the square root of the average of all the misclassification errors. It is a very commonly used performance metric for numeric predictions and can be calculated using

(10) $$\begin{array}{c} RMSE=\sqrt{\frac{\sum_{i=1}^{N}{\left(d_{i}-z_{i}\right)}^{2}}{N}}, \end{array}$$

where *N* shows the size of data, *d*_*i*_ shows the actual label of a data instance, and *z*_*i*_ indicates the predicted label of a patient.

In ROC analysis, a threshold is set to differentiate between the actual data and noisy data, that is, misclassifications. ROC curve is used to show the effect of changing thresholds on the detection accuracy. If the threshold is set to a high value, then most of the correct detection instances are missed while a low threshold may incorporate a lot of false alarms [41].

### 4.3. Parameter Evaluation

The arrhythmia detection performance is evaluated using different parameters to select the most suitable parameters for classification. SVM methods are applied on the dataset with varying parameters and results are evaluated using accuracy, kappa statistics, and RMSE to highlight the most promising parameter for classification. First, the classification model is implemented using different train/test split options to evaluate the system's accuracy. The results of SVM methods using five different split options are shown in Table 1, which shows that OAO method gives the most accurate results and achieves the highest accuracy when 90% of the overall dataset is placed under training set and 10% of the data is used for testing. Data overfitting occurs, when large amount of data is used for training, so a standard 80/20 data split is used for further implementation of the proposed model. It is evident that OAO approach resulted in the highest testing accuracy rate but overall obtaining a very high accuracy is a challenging task. This could be due to the existence of a single class having the highest proportion of data instances; therefore, to ensure good training of the model, a uniform data distribution is needed.

**Figure 3:**
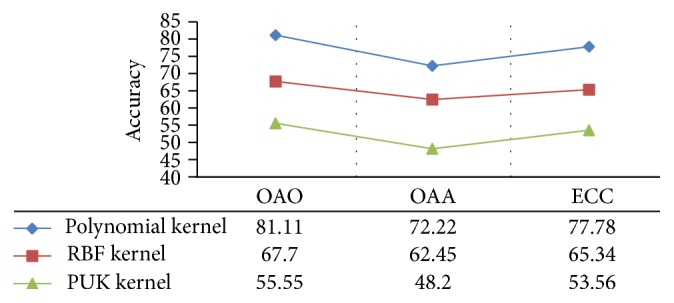
A comparison of classification performances for different SVM kernels.

The results for the SVM based methods are further evaluated using variants of kernel parameters. The best results are obtained when a polynomial kernel is used with SVM for classification with a gamma value of 0.001. The results are cross-checked with other kernel approaches including radial basis function (RBF) based kernel and Pearson-VII (PUK) function based universal kernel. The results of different kernels are compared and are shown in Figure 3, where the highest accuracy rate is achieved using a polynomial kernel.

Similarly, the model with the applied feature selection technique is tested on some other machine learning classifiers including RF, NB, MLP, and KNN for comparisons and the results are evaluated for the test data using three performance measures mentioned earlier. The results shown for RF also performed well and the results for RF are very close to SVM, that is, 80%. As RF is a knowledge based ensemble tree method and decision trees perform best for multiclass classification problem by automated handling of the missing data and outliers in training and testing phase, for RF, a total of 1000 trees are built for each iteration. MLP is a neural network approach particularly designed for multiclass problems; hence it also yields good classification accuracy. As MLP is not a knowledge based ensemble technique like RF, therefore it provides marginally less accurate results but still outperforms KNN approach when used with feature selection.

Table 2 shows that SVM outperformed all other classifiers with an accuracy of 81.11%, since SVM is a strong classifier, which can deliver high performance results even when a small learning dataset is used. The results of SVM are mentioned for OAO approach and a polynomial kernel is used with a tolerance parameter of 0.001. The other classifiers are also implemented using pairwise coupling as it is a multiclass classification problem.

RF also performed well and the results for RF are very close to SVM, that is, 80%. As RF is a knowledge based ensemble tree method and decision trees perform best for multiclass classification problem by automated handling of the missing data and outliers in training and testing phase, for RF, a total of 1000 trees are built for each iteration. MLP is a neural network approach particularly designed for multiclass problems; hence it also yields good classification accuracy. As MLP is not a knowledge based ensemble technique like RF, therefore it provides marginally less accurate results but still outperforms KNN approach when used with feature selection.

**Figure 4:**
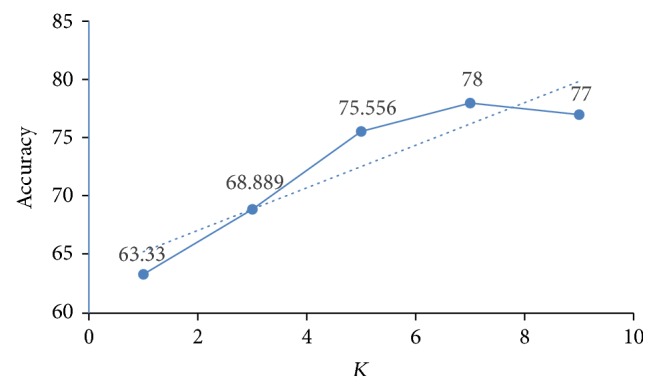
Accuracy of KNN with varying *K* values.

The results of KNN are collected in five iterations by varying the value of *K*, that is, the number of neighbors in each iteration. The value of *k* varies in the range of 1 to 9, where only odd values are selected to avoid any conflict in selecting the majority output class. The results of KNN in terms of accuracy for different “*K*” values are depicted in Figure 4, which shows that the classifier gives best accuracy rate when *K* equals 7. KNN is tested using one-against-all approach of multiclass classification and a distance weighting approach based on the Euclidean distance is used for calculating the distance of nearest neighbors. The results are cross-validated and an average accuracy of 77.78% is achieved which is higher as compared to the state-of-the-art methods but still a bit lower when compared with other SVM approaches used in this proposed work.

Computationally, KNN is easy to run even for 1000 iterations and using high folds in cross-validation methods. NB also provides similar results when a kernel estimator is used as a parameter for implementation. These classifiers are also tested on the whole dataset without applying any feature selection technique with the same set of parameters to evaluate the performance of our proposed model. The results showed that, without feature selection, NB classifier gives the best results. The accuracy is still less than the proposed model with feature selection technique and hence the proposed system proves to be a good contribution to the field of disease diagnosis and decision-making. The results of classifiers are also evaluated using kappa statistics and RMSE. The kappa statistics values near 1 show the best results in terms of correct distribution among classes. Similarly, RMSE shows better results when reported closest to 0. From the results of Table 2, it is seen that the best prediction results in terms of kappa statistics and RMSE are obtained when SVM OAO method is employed for predicting the correct arrhythmia disease type.

Similarly, the model with the applied feature selection technique is tested on some other machine learning classifiers including RF, NB, MLP, and KNN for comparisons and the results are evaluated for the test data using three performance measures mentioned earlier. The results are shown in Table 3 and show that SVM outperformed all other classifiers with an accuracy of 81.11%, since SVM is a strong classifier, which can deliver high performance results even when a small learning dataset is used. The results of SVM are mentioned for OAO approach and a polynomial kernel is used with a tolerance parameter of 0.001. The other classifiers are also implemented using pairwise coupling as it is a multiclass classification problem. RF also performed well and the results for RF are very close to SVM, that is, 80%. As RF is a knowledge based ensemble tree method and decision trees perform best for multiclass classification problem by automated handling of the missing data and outliers in training and testing phase, for RF, a total of 1000 trees are built for each iteration. It is to be noted that, here, RF is used as a classifier to solve a multiclass classification problem of arrhythmia diagnosis. MLP is a neural network approach particularly designed for multiclass problems; hence it also yields good classification accuracy. As MLP is not a knowledge based ensemble technique like RF, therefore it provides marginally less accurate results but still outperforms KNN approach when used with feature selection. TSVM approach is also tested for arrhythmia dataset and an accuracy of 49.74% is achieved. The parameters are selected such that* c*1 and* c*2 are picked from the set of {−4, −2,0, 1,2, 4,6, 8}, a polynomial kernel is used with *p* parameters selected from the set {−4, −2,0, 2,4, 6,8}, and the value of tolerance parameter is selected from the set of {0.1,0.3,0.5,0.7,0.9}. The result shows that TSVM does not give optimal results, when used with wrapper feature selection methods as compared to other methods.

Another set of experiments is conducted in which the dataset is tested with SVM based OAO multiclassifier model by varying the feature selection techniques. This experiment is performed with 80% of the data used for training, and results of the experiment are evaluated using 20% of the data kept for testing. The performance of our proposed feature selection technique is compared with three well-known and widely used methods including principal component analysis (PCA), correlation based feature selection (CFS), and information gain based feature selection (IGFS). The results are evaluated using three different parameters and are shown in Table 3, which shows that the wrapper technique for feature selection provides the highest classification accuracy, that is, 81.11%. The highest accuracy shows that the wrapper method selects the most important attributes that contributed significantly to the classification process.

The PCA based method selected 79 attributes and achieved an average classification accuracy of 76.67% which is higher as compared to CFS and IGFS but still less than the accuracy achieved by the proposed wrapper technique. The basic concept of using PCA for feature selection is to select features based on the magnitude of their coefficients starting from the largest value to the smallest value. The problem in using PCA is that only a linear relationship of features is considered and the multivariate nature of dataset is not considered. CFS selects a total of 91 input features out of 279 and gives the lowest accuracy rate of 60% as compared to other techniques. It is one of the popular techniques used for selecting the most significant attributes and is usually referred to as a Pearson correlation coefficient in statistical analysis. In this approach, the correlation among the input attributes and the resultant variable is calculated and the attributes show correlations closer to −1 or +1. All other attributes with a lower correlation closer to 0 are ignored or removed. A cut-off parameter with a value of 0.09 is selected in this study. Similarly, IGFS gives an accuracy of 68.89%, when used with SVM based OAO method for classifying arrhythmia. It is calculated using an entropy measure for each input attribute against the output variable. The resultant entropy values range from 0 to 1, where 0 shows no information and 1 shows the highest significant data. Therefore, attributes with higher information gain are selected for classification and others with a lower gain are removed. A threshold of 0.01 is set for selecting the attributes and a total of 23 attributes are selected.

**Figure 5:**
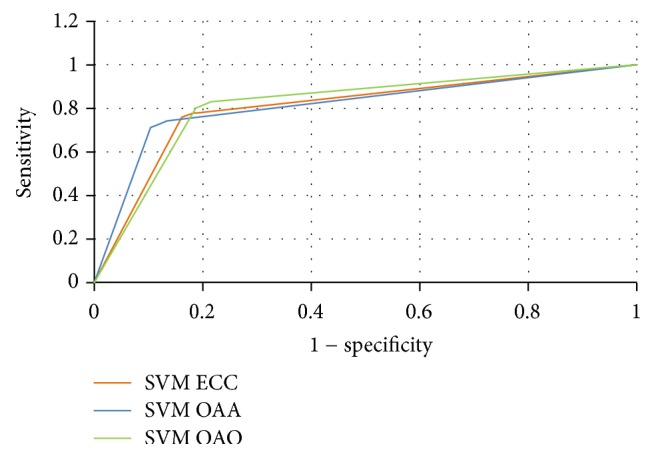
ROC curve for different SVM methods used in this study.

The results of the arrhythmia classification are also presented with ROC curve as shown in Figure 5. The ROC curve plots the true positive rates and the false positive rates of the specified classification algorithms. In this research, a multiclass classification problem is addressed for classifying arrhythmia in 16 classes and the ROC curve is obtained by taking an average of the output classes. Each point located on the curve line illustrates a pair of sensitivity and specificity values calculated for a setting of decision threshold. A good classification method has an ROC curve that passes through the top left corner of the graph. The ROC curve drawn for OAO is closer to 1 representing more accurate results for that method. Similarly, the other two ROC curves drawn for AOA and ECC exhibit a reasonable performance rate for classification.

### 4.4. Comparison with State of the Art

To evaluate the proposed method, results are compared with the state-of-the-art methods for the UCI-arrhythmia dataset. As shown in Table 4, the proposed model shows remarkable performance as compared with recent methods. Mitra and Samanta [16] used correlation based selection feature selection (CFS) approach with linear forward search and selected 94 features. They achieved 87.71% average accuracy by using incremental backpropagation neural network (IBPLN) and Levenberg-Marquardt (LM) for classification. Jadhav et al. [15] achieved 78.89% average accuracy on UCI-arrhythmia dataset by using modular neural network with three layers. The missing values are replaced with the closest values for the related attribute from the same class and the proposed model is evaluated by dividing the dataset into different groups and the best accuracy reported was for 90-10 training and test split.

Batra and Jawa [18] used a combination of SVM and grant boosting for classification with radial kernel and selected 60 features using grant boosting model. In [26], Namsrai et al. formulated an ensemble method for classification by using a voting approach on selected features. SVM and Naïve Bayes classifier shows best result when applied on 70-30 training/testing dataset split. In [24], feature selection method consisted of two parts, that is, filter part and wrapper part, and the best accuracy is reported using SVM and KNN classifier with 20-fold cross-validation. In [13], a 3-fold cross-validation technique is used, achieving the best accuracy for MLP/NN classifier. Gao et al. [14] compared their proposed model on Naïve Bayes, decision trees, logistic regression, and RBF networks with neural networks. The neural network approach showed the best performance as compared to other classifiers.

## 5. Conclusion and Future Work

This paper proposes a method for multiclass classification of arrhythmia using ECG records with three different SVM based approaches. A wrapper based feature selection method is proposed for selecting the most significant features to reduce the dimensions of data. The data is also normalized to avoid conflicts occurring due to the presence of binary values. SVM based methods including one-against-all, one-against-one, and error correcting codes are then applied on the normalized data to detect the presence or absence of disease and classify the records into one of the sixteen given classes. The feature selection, preprocessing, and classification techniques have produced a combination which provides promising results for disease classification. The classification results indicate that one-against-one method is best suited for classification on the ECG dataset taken from UCI repository. Some other classifiers are also implemented using the proposed WFS and normalization approaches and the results show that the proposed method outperforms other state-of-the-art methods employed for classification of arrhythmia using similar dataset. The potential of the one-against-one method suggests that it can be improvised to be used on other disease datasets as well.

## Figures and Tables

**Table 1:** Classification accuracy of SVM methods for different data splits.

SVM methods	Accuracy with respect to dataset splits percentages				
	50/50	60/40	70/30	80/20	90/10
OAA	60.18	60.22	67.65	72.22	80.00
OAO	73.45	74.59	77.20	81.11	92.07
ECC	69.91	71.82	76.47	77.78	88.89

**Table 2:** Performance comparison of various classifiers with and without feature selection.

Feature selection	Performance metrics	Machine learning algorithms				
		SVM	NB	MLP	RF	KNN
Yes	Accuracy (%)	81.11	77.78	78	80	77.78
	Kappa statistics	0.72	0.62	0.65	0.66	0.61
	Root mean square error	0.17	0.26	0.27	0.25	0.26

No	Accuracy (%)	64.40	75.50	73.00	72.22	62.22
	Kappa statistics	0.52	0.58	0.47	0.47	0.21
	Root mean square error	0.22	0.19	0.18	0.18	0.20

**Table 3:** Performance comparison of feature selection methods.

Feature selection method	Number of selected features	Accuracy (%)	Kappa statistics	RMSE
PCA	79	76.67	0.67	0.18
IGFS	23	68.89	0.55	0.20
Correlation based selection	91	60.00	0.45	0.22
Wrapper method	94	81.11	0.72	0.17

**Table 4:** Comparison of the proposed model with state-of-the-art methods.

Method	Train-test split	Number of selected features	Accuracy (%)
*Proposed*	*90-10*	*94*	*92.07%*
IBPLN + LM [16]	68-32	18	87.71%
Modular NN [15]	90-10	198	78.89%
SVM [18]	75-25	60	84%
NB [26]	70-30	205	70.50%
KNN [24]	20-fold CV	148	73.80%
MLP NN [13]	3-fold CV	-	88.24%
NN [14]	90-10	79	76.67%
